# Supplementary material for: B-Cell Activation Biomarkers in Salivary Glands Are Related to Lymphomagenesis in Primary Sjögren’s Disease: A Pilot Monocentric Exploratory Study
Source: Int J Mol Sci. 2024 Mar 13;25(6):3259. doi: 10.3390/ijms25063259 (PMC10969818; doi:10.3390/ijms25063259)
Supplement: Supplementary file 1 [file ijms-25-03259-s001.zip › ijms-2891004-supplementary.pdf]

Supplementary Materials

B-Cell Activation Biomarkers in Salivary Glands Are Related to Lymphomagenesis in Primary Sjögren's Disease: A Pilot Monocentric Exploratory Study

Dario Bruno [1,2], Barbara Tolusso [3], Gianmarco Lugli [4], Clara Di Mario [3], Luca Petricca [5], Simone Perniola [1], Laura Bui [6], Roberta Benvenuto [6], Gianfranco Ferraccioli [7], Stefano Alivernini [3,5,7,*] and Elisa Gremese [1,3,7,*]

[1] Clinical Immunology Unit, Fondazione Policlinico Universitario A. Gemelli-IRCCS, 00168 Rome, Italy; dariobrunomd@gmail.com (D.B.); simone.perniola@guest.policlinicogemelli.it (S.P.)
[2] Department of Medicine, University of Verona, 37129 Verona, Italy
[3] Immunology Core Facility, Gemelli Science and Technology Park, Fondazione Policlinico Universitario A. Gemelli-IRCCS, 00168 Rome, Italy; barbara.tolusso@policlinicogemelli.it (B.T.); dimarioclara@gmail.com (C.D.M.)
[4] Rare Disease Unit, Meyer Children's Hospital IRCCS, 50139 Florence, Italy; gianmarco.lugli@meyer.it
[5] Rheumatology Division, Fondazione Policlinico Universitario A. Gemelli-IRCCS, 00168 Rome, Italy; luca.petricca@policlinicogemelli.it
[6] Institute of Pathology, Fondazione Policlinico Universitario A. Gemelli IRCCS, 00168 Rome, Italy; laura.bui@policlinicogemelli.it (L.B.); roberta.benvenuto@policlinicogemelli.it (R.B.)
[7] Department of Internal Medicine, Catholic University of the Sacred Heart, 00168 Rome, Italy; gianfranco.ferraccioli@unicatt.it
* Correspondence: stefano.alivernini@unicatt.it (S.A.); elisa.gremese@policlinicogemelli.it (E.G.)

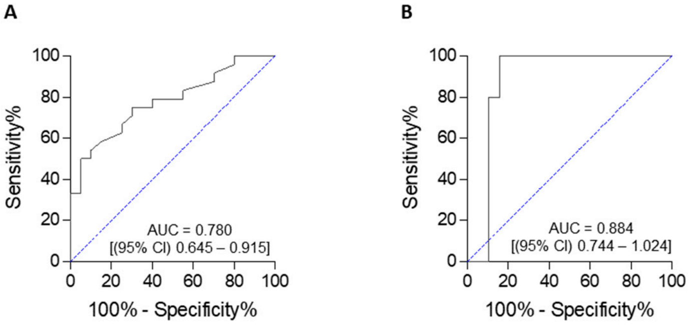

Supplementary Figure S1. (**A**,**B**). ROC curve analysis showing the power of LSG miR-155 expression (fold-change) in distinguishing patients with sicca syndrome from those with primary Sjögren's disease (pSS) (**A**) and identifying patients with pSS who developed NHL (**B**). ROC, receiver operating characteristic curve; LSG, labial salivary glands; miR-155, MicroRNA 155; pSS, primary Sjögren's syndrome; NHL, Non-Hodgkin's lymphoma.
